# Symbiont-derived β-1,3-glucanases in a social insect: mutualism beyond nutrition

**DOI:** 10.3389/fmicb.2014.00607

**Published:** 2014-11-21

**Authors:** Rebeca B. Rosengaus, Kelley F. Schultheis, Alla Yalonetskaya, Mark S. Bulmer, William S. DuComb, Ryan W. Benson, John P. Thottam, Veronica Godoy-Carter

**Affiliations:** ^1^Department of Marine and Environmental Sciences, Northeastern UniversityBoston, MA, USA; ^2^Department of Biological Sciences, Towson UniversityTowson, MD, USA; ^3^Department of Biology, Northeastern UniversityBoston, MA, USA

**Keywords:** gut protozoa, disease resistance, β-1, 3-glucanases, termites, social immunity, mycosis

## Abstract

Termites have had a long co-evolutionary history with prokaryotic and eukaryotic gut microbes. Historically, the role of these anaerobic obligate symbionts has been attributed to the nutritional welfare of the host. We provide evidence that protozoa (and/or their associated bacteria) colonizing the hindgut of the dampwood termite *Zootermopsis angusticollis,* synthesize multiple functional β-1,3-glucanases, enzymes known for breaking down β-1,3-glucans, the main component of fungal cell walls. These enzymes, we propose, may help in both digestion of ingested fungal hyphae and protection against invasion by fungal pathogens. This research points to an additional novel role for the mutualistic hindgut microbial consortia of termites, an association that may extend beyond lignocellulolytic activity and nitrogen fixation to include a reduction in the risks of mycosis at both the individual- and colony-levels while nesting in and feeding on microbial-rich decayed wood.

## INTRODUCTION

The impacts of mutualistic gut microorganisms may extend beyond the confines of the digestive system, affecting not only aspects related to the animal’s nutrition, weight control, and metabolism, but also behavior, neuronal development, learning, and memory (Grenham et al., 2011; Kinross et al., 2011). Recently, the study of gut microbiomes and their repercussion on vertebrate and invertebrate host longevity, overall health status and ability to fight systemic disease has received much attention (Grenham et al., 2011; Kinross et al., 2011). Insects are no exception; their diverse microbiota has been implicated in their resistance against pathogens and parasites (Scarborough et al., 2005; Moran, 2006; Haine, 2008; Ryu et al., 2008; Feldhaar, 2011; Mattila et al., 2012; Parker et al., 2013; Oliver et al., 2014). Given the long coevolutionary history between termites and their diverse gut microbiota (likely established at least ∼100 million years ago, Wier et al., 2002; Poinar, 2009), this social insect group is an excellent candidate to study the nature and dynamics of this mutualism at both the individual- and colony-levels.

The dampwood termite *Zootermopsis angusticollis* nests at high densities in homeothermic and humid microclimatic conditions within their decomposed wood. These settings favor high risks of infection, particularly by entomopathogenic fungi (Blackwell and Rossi, 1986; Rosengaus et al., 2011) which are known to affect the individual’s and colony’s survival (Rosengaus et al., 1998a; Rosengaus and Traniello, 2001). One common soil fungus, *Metarhizium anisopliae*, has received significant attention as a potential biological control agent of termites (Chouvenc et al., 2011). However, this entomopathogen has had limited success in the control of pest species in the field (Rath, 2000; Chouvenc et al., 2011). One possible reason for such failure involves the refractory nature of the termite’s gut against fungal infection following conidia ingestion during and after bouts of mutual grooming between infected nestmates (Chouvenc et al., 2009; Rosengaus et al., 2011). Thus, we asked what is (are) the compound(s) along the termite’s alimentary canal responsible for the reduction of fungal conidia viability?

Several candidate antifungal compounds exist, including norharmane which is likely produced by Actinomycete bacteria (Siderhurst et al., 2005; Chouvenc et al., 2008). While norharmane is toxic against several organisms (Rivas et al., 1999), *in vitro* tests at termite physiological concentrations showed a limited effect on *M. anisopliae* mycelial growth (Chouvenc et al., 2008). Alternatively, termicins in salivary glands and other termite tissues have been shown to have powerful antifungal properties (Lamberty et al., 2001; Hamilton and Bulmer, 2012). These proteins could be swallowed along with the groomed-off conidia and/or may be synthesized in the gut itself, which could explain why the termite’s alimentary canal appears inhospitable to fungus. Another possible reason for the refractory nature of the termite gut may include the presence of β-1,3 glucanases (β-1,3GLUs) known for breaking-down β-1,3 glucans, the main components of fungal cell walls (Brown and Gordon, 2005). In termites, these functional enzymes were first reported in *Nasutitermes* (Bulmer et al., 2009) and subsequently in *Reticulitermes* (Hamilton et al., 2011; Hamilton and Bulmer, 2012). Termites are classified as “lower” or “higher” termites based on the type of symbionts found in their hindguts. Notably, *Nasutitermes* (a “higher” termite harboring only bacteria in its hindgut; Ohkuma and Brune, 2011) has the same two β-1,3GLUs in both the body (minus the gut) and the gut, whereas *Reticulitermes* (a “lower” termite with its hindgut colonized by both protozoa and bacteria, Brune, 2014) has two β-1,3GLUs in its body and multiple additional β-1,3GLUs in its gut (Bulmer et al., 2009, 2010; Hamilton et al., 2011; Hamilton and Bulmer, 2012). Such differences in the gut β-1,3GLU profiles between the “higher” (lacking protozoa symbionts) and “lower” termites (associated with an abundant and varied hindgut protozoa) led us to hypothesize that the multiple β-1,3GLUs of *Reticulitermes* guts (and perhaps those of other “lower” termites) are synthesized by its protozoa community.

We used the “lower” dampwood termite *Z. angusticollis* to establish whether β-1,3GLUs also exist in this species and systematically test the source of origin and function of such additional enzymes. Through a series of experiments using chromogenic gels for the visualization of functional β-1,3GLUs of different termite tissues, as well as fractionation, *ex vivo* protozoa culturing and defaunation assays, we show that the protozoa (and/or its associated bacteria) colonizing the hindgut of *Z. angusticollis* are the most likely candidates for the synthesis of these enzymes. Moreover, *in vitro*, enzyme-inhibition and *in vivo* experiments point to the possibility that both the termite-derived and symbiont-derived β-1,3GLUs have fungistatic activity which may translate into lower susceptibility to mycosis for the termite hosts. Hence, a potential novel role for the mutualistic association between termites and their hindgut microbial consortia is proposed, one that goes beyond their historically accepted role in ligno-cellulose digestion and nitrogen metabolism (Brune, 2014).

## MATERIALS AND METHODS

### TERMITE COLLECTION

A total of eight colonies of the dampwood termite, *Z. angusticollis*, were collected from the Huddart Park (San Mateo County) and the Redwood East Bay Regional Park in Oakland, CA, USA. These stock colonies, maintained in our USDA permitted containment room at 25°C inside closed plastic containers, were the source of insects for all of the different experiments described below. Colonies were given water and birch wood as food *ad libitum* and the termites were used within the same year they were collected from the field.

### GUT DISSECTIONS AND PREPARATION OF TERMITE EXTRACTS

Termites (nymphs and pseudergates, also known as false workers) were cold immobilized and their entire guts were pulled through the anus by using sterile fine tipped forceps. Crude extracts of different termite tissues [degutted bodies, entire guts, different gut regions (fore, mid, and hindgut) and liquid feces] were first prepared by homogenizing the respective sample in acetate buffer (0.2 M, pH 5.0, 4 μL/termite) inside a Biomasher (pore size 80–145 μm; Cartagen) according to Bulmer et al. (2009). Biomashers were then centrifuged for 1 min at 4°C and 14,000 × *g*. To control for differences in the size of the various tissues amongst termites, extracts were standardized as a function of total protein content by running a Quick Start Bradford Protein Assay (Bio-Rad, USA). The adjusted extract volumes were then diluted with Native Sample Buffer (2:1) and subsequently loaded in chromogenic gels for the visualization of β-1,3GLUs (see below).

### VISUALIZATION OF β-1,3GLUs

**FIGURE 1:**
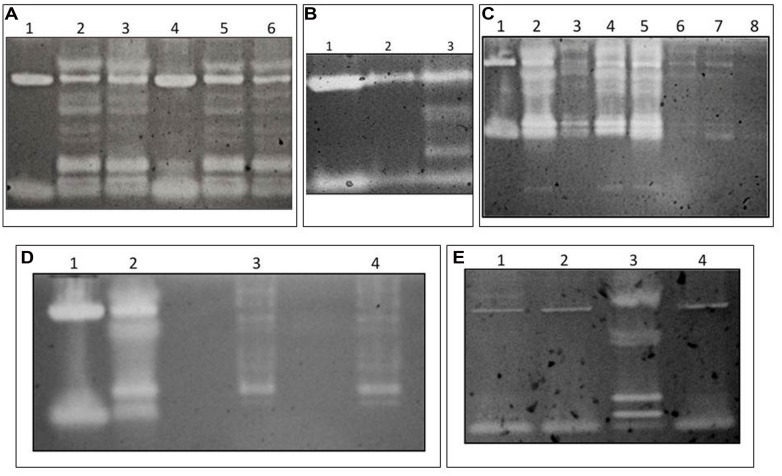
**Evidence for β-1,3GLU activity in *Zootermopsis angusticollis* extracts run in CM-Curdlan-RBB gels. (A)** Extracts of gutted bodies (lanes 1 and 4), dissected guts (lanes 2 and 5) and expressed liquid feces (lanes 3 and 6). **(B)** β*-*1,3GLU activity in extracts of the foregut (lane 1), midgut (lane 2) and hindgut (lane 3). The final volume loaded per lane was standardized to a total protein content of 1.5 mg/mL. **(C)** Results from the fractionation procedure depicted in Supplementary Figure 2, degutted body from a faunated termite (lane 1), faunated gut (lane 2), washed faunated gut (lane 3), protozoa and associated bacteria (lanes 4 and 5), free-living lumen bacteria (lanes 6 and 7), cell-free supernatant (lane 8). **(D)** Results of *ex vivo* culture of termite hindgut symbionts. Extracts of the degutted termite body (lane 1), a faunated gut (lane 2), and extracts of *ex vivo* cultured protozoa (lanes 3 and 4). The similar β*-*1,3GLU profile between the *ex vivo* cultures and the gut of faunated controls [after the pressurization treatment with no oxygenation lane 2)] provide further support that the multiple hindgut β*-*1,3GLUs are of protozoa origin. **(E)** Lack of symbiont-derived β*-*1,3GLU activity in guts of defaunated (oxygenated) termites (lane 1) relative to the presence of activity in faunated control (pressurized only) termites (lane 3). Note that the two clearing zones of the host-derived (body minus gut) β*-*1,3GLUs in lane 2 are intact in spite the oxygenation treatment. The β*-*1,3GLUs profile of lane 2 is comparable to that of the degutted body of a pressurized (no added oxygen) control (compare lanes 2 and 4). Hence, oxygenation only eliminates the symbiont-derived β*-*1,3GLUs.

To visualize β-1,3GLUs in the various termite tissue extracts, Carboxymethyl Curdlan Remazol Brilliant Blue (Loewe Biochemica) gels were prepared following previously published methods (Kalix and Buchenauer, 2005; Bulmer et al., 2009). Briefly, the curdlan gel matrix consists of β-(1,3)-linked glucose residues covalently bound to the Ramazol blue dye. If tissue extracts contain β-1,3GLUs activity, the enzymes, following migration within the gel, digest the gel matrix and release the dye after gel rinsing. Therefore, the presence of active β-1,3GLUs in our crude tissue extracts can be visualized as light clearing zones in the gel (**Figures 1A–E**). The gels were run at 50 V for 20 min, and then run at 150 V until completion (approximately 60 min). The gel was then incubated with a 100 mM NaAc buffer (pH = 5.0) for 18–20 h on a shaking platform (50 rpm). All gels were rinsed with deionized water after incubation. Gels were photographed using a Kodak Gel Logic 10 camera.

### GUT FRACTIONATION

To establish whether the sources of the multiple β*-*1,3GLUs were the protozoa, their associated bacteria (ecto and endosymbionts) or the free-living bacteria found in the lumen of the termite’s alimentary canal (Brune, 2006), samples were separated based on density (Morgavi et al., 1994) and subsequently run on CM-curdlan-RBB gels. The contents of two termite guts were transferred to 1000 μL of sterile Trager U solution (Trager, 1934). One of the emptied guts was gently washed in sterile Trager U and rinsed with a syringe in order to test for β*-*1,3GLU activity of the rinsed gut tissue. The gut contents of the second dissected gut were first centrifuged at a low speed (4°C, 550 × *g*, 5 min) to pellet down the protozoa and associated bacteria, while leaving the free-living bacteria in suspension (Supplementary Figure 1). The supernatant was then removed and placed into a separate tube and centrifuged at a high speed (4°C, 20 800 × *g*, 30 min) to collect the lumen free-living bacterial pellet (Supplementary Figure 1). The supernatant from the second spin was saved as a cell-free fraction (Supplementary Figure 1). All symbiont fractions were examined microscopically for presence of bacteria and/or protozoa. The lumen bacteria fractions were determined to be protist-free, while the mainly protozoa fraction still contained visible bacteria. Native sample buffer (Bio-Rad) was added to the protozoa/bacteria pellet, free-living lumen bacterial pellet, and the cell free fraction prior to loading in the CM-curdlan-RBB gel.

### *EX VIVO* SYMBIONT CULTURES

To test if the multiple β-1,3GLUs visualized from the hindgut (**Figure 1B**) were of symbiont-origin, the symbionts were cultured *ex vivo.* The protocol from Coleman (1991) was followed with two main modifications: the gut symbionts were obtained from the ruptured termite hindgut by using a sterile gel loading pipette tip and symbionts were cultured in glass tubes with screw top caps. Capped inoculated culture tubes were incubated at 27°C and sub-cultured after 2–3 weeks by inoculating 2 mL of the original culture into 10 mL of new culture medium. Cultures were checked periodically using a hemocytometer in order to quantify protozoa numbers. Twelve-day-old culture fluid was concentrated by modifying the protocol from (Yamin, 1978). Briefly, three to four mL of culture fluid were layered over 6 mL of chilled 20% Ficoll 400 (Sigma Chemical Co) solution in a 10 mL culture tube. These samples were subsequently centrifuged at 4°C and 900 × *g* for 20 min. Protozoa along with associated bacteria were collected from the interface. To ensure a clean sample, the interface fluid was re-centrifuged (4°C, 8000 × *g*, 30 min) and the supernatant removed and discarded. The remaining pellet was resuspended in 250 μL of Trager U solution (Trager, 1934) and again centrifuged (4°C, 8000 × *g*, 10 min). The supernatant was again discarded and the remaining pellet was re-suspended a final time with 50 μL of Trager U. Ten microliters of the final suspension were examined under the microscope (Nikon Eclipse E400, 400× magnification) to confirm presence of protozoa. Twelve-day-old fractionated *ex vivo* samples were then prepared for use in chromogenic electrophoresis as described earlier.

### DEFAUNATION PROCEDURE

To further test whether the termite’s gut microbiome synthesized β-1,3GLUs, we eliminated the anaerobic microbes by adapting an oxygenation system from Cleveland (1925). Ten nymphs and pseudergates were randomly selected and placed inside open-ended 15 mL plastic culture tubes. Tubes were lined with a strip of paper towel moistened with 300 μL of sterile water. Both sides of the tubes were then plugged with moistened cotton-balls and placed into a steel pressure canister (Sure-Shot Atomizer^©^). The canister containing the termites targeted for defaunation was subsequently connected to an oxygen tank and the canister was first flushed with oxygen for 60 s to remove residual air. The steel canister was then sealed as oxygen from the tank continued to flow into it, until a pressure of 40 psi was reached. The termites remained under high pressure oxygen for 1 h and then the canister was depressurized (but not opened). The termites were kept under these concentrated oxygen levels (≈97%) for 24 h, post depressurization. Control faunated termites were subjected to identical pressurization method as those of defaunated nestmates with the exception that they were not exposed to additional oxygen, but instead exposed only to pressurized air. After such treatments, one to two termites from each group were randomly selected to confirm the effectiveness of the oxygenation treatment in causing defaunation relative to termites in the control treatment. Confirmation was achieved by examining 10 μL of the hindgut fluid of a dissected termite on a hemocytometer using a compound light microscope (Nikon Eclipse E400) at 400× magnification and enumerating the number of intact protozoa in the sample (Supplementary Figure 2).

### *IN VITRO* ANTIFUNGAL ASSAY

Two different methods were used to test *in vitro* the effect of β-1,3GLUs on conidia viability. We first quantified the long-lasting effects (at 96 h of plating) of β-1,3GLUs on conidia viability by enumerating colony forming units (CFUs). The effects on conidia viability earlier during fungus development were also tested by quantifying conidia germination at 18 h following plating. Although the protocols for each of these methods differ (see below and Supplementary Figure 3), both provide accurate measurement of antifungal properties of termite extracts (and their accompanying β-1,3GLU s) at different stages of fungus development (Supplementary Figure 3). Long-lasting effects can be traced back to effects during the early stages of fungus development: fewer CFUs four days post-plating result from lower germination rates18 h post-plating.

**FIGURE 2:**
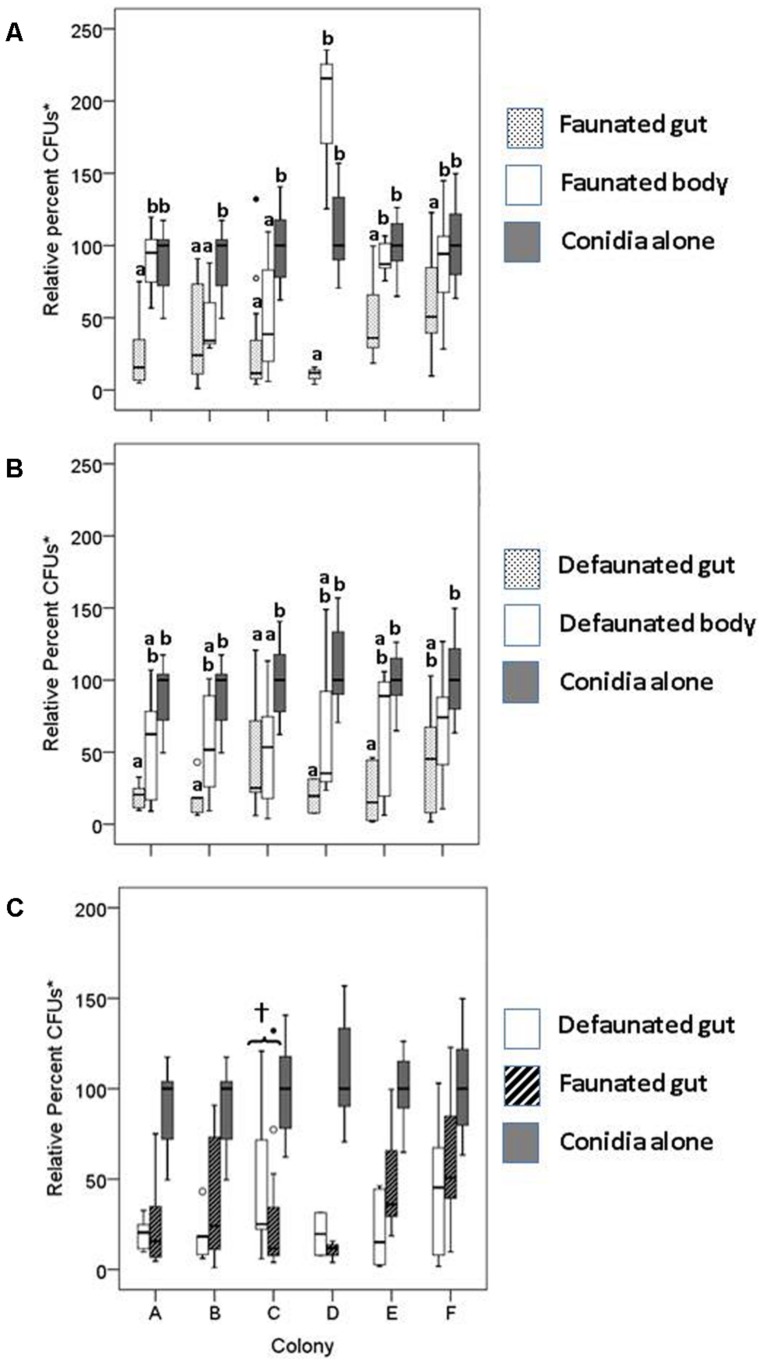
Relative percent colony forming units (CFU)* of conidia mixed with extracts of faunated **(A)** and defaunated **(B)** termites from six different stock colonies. (C) Conidia viability after incubation with gut extracts from faunated (pressurized only) and defaunated (oxygenated and pressurized) termites. Each boxplot shows the median value and interquartile range. The outliers, identified by small circles, included cases with values between 1.5 and 3 box lengths from the upper edge of the box. Bars with the same letter are not significantly different (*p* > 0.017) in pairwise comparisons within a colony (by MW test adjusted with Bonferroni correction, SPSS). In **(C)**, the only significant difference in conidia viability between defaunated and faunated guts was observed for colony C (†). None of the other pairwise comparisons within colonies was statistically significant. *CFUs as percent of the median of the control treatment.

To test the long-lasting fungistatic properties of termite tissue, extracts of both faunated and defaunated termites (as described above) were incubated with fungal conidia, then plated and fungal CFUs enumerated, following the methods outlined below. Immediately after the pressure only (faunated controls) and pressure plus oxygen (defaunated) treatments, termites were placed into glass dishes with a plaster of Paris substrate that was moistened with 1000 μL of sterile water and maintained there for 24 h. The plaster permitted termites to be kept under high moisture while eliminating feeding on cellulose material (filter paper or wood). Hence, both defaunated and faunated treatments experienced the same degree of starvation and therefore we controlled for the effect of nutritional status on antifungal properties of the gut. Throughout the 24 h on the moistened plaster, any residual glucanases from the hindgut symbionts were likely flushed from the digestive system of the defaunated termites. Extracts of these termite guts were prepared and then incubated with 10 μL of a 50 mg/mL solution of ampicillin (to control bacterial overgrowth) and 10 μL of a 1 × 10^4^ conidia/mL suspension of *M. anisopliae* for 24 h at 25°C while gently shaken at 50 rpm. Conidia initiate germination only after plating onto the agar medium and hence, we are certain no germination occurred during the incubation of conidia with the termite extracts. Control samples (lacking termite extracts) were created with 10 μL NaAc buffer, 10 μL sterile distilled water, 10 μL of ampicillin, and 10 μL of a 1 × 10^4^ conidia/mL suspension of *M. anisopliae*. These control samples were also incubated and shaken alongside the experimental groups. After 24 h of incubation, 60 μL of sterile distilled water was added to each tube and 100 μL of the resulting solution was plated using sterile glass beads onto PDA plates (100 mm × 15 mm) supplemented with 50 μg/mL of ampicillin and incubated at 25°C for 96 h after which CFUs were counted (**Figure 2**, Supplementary Figures 3A,B).

### *IN VITRO* ANTIFUNGAL ASSAY, RESCUE EFFECT OF D-GLUCONO1,5-LACTONE (GDL)

In a parallel study and to establish if fungistatic effects of termite tissue extracts affected fungus viability earlier during its development, we quantified conidia germination rates. To this end, 5 μL of a 20 mM solution of the glucanase inhibitor GDL (Bulmer et al., 2009) were added to the tissue extracts. If β*-*1,3GLUs had fungistatic effect on conidia, then blocking the β*-*1,3GLUs active sites should enhance or completely rescue fungal viability relative to un-blocked β*-*1,3GLUs. Entire guts (fore-, mid-, hingut, rectum) of 11 nymphs were placed into a Biomasher (Cartagen) fitted with a filter (pore size 80–145 μm). The corresponding degutted bodies were placed into a second Biomasher. To each tube, 60 μL of sterile distilled water was added. The contents of the tubes were then ground with a pestle and centrifuged (4°C, 14,000 × *g*, 1 min). The liquid flow-through was transferred to Costar Spin-X 0.22 μm (Corning) filters (20 μL each filter). Twenty microliter of 200 mM NaAc (pH 5.0) was then added to the filter and centrifuged (4°C, 12,000 × *g*, 4 min) to filter out any bacteria contaminants and remaining termite tissue. The flow-through of the samples was then pooled and 4 μL were observed under a microscope (400×) to determine if there was any termite tissue. If termite tissue was present, repeated filtration continued until the flow-through was clean. The filtrate (20 μL) was mixed with 10 μL of ampicillin (50 μg/mL), 10 μL of *M. anisopliae* (10^8^ conidia/mL), and 5 μL of GDL (100 mM; or distilled water for no GDL control).

**FIGURE 3:**
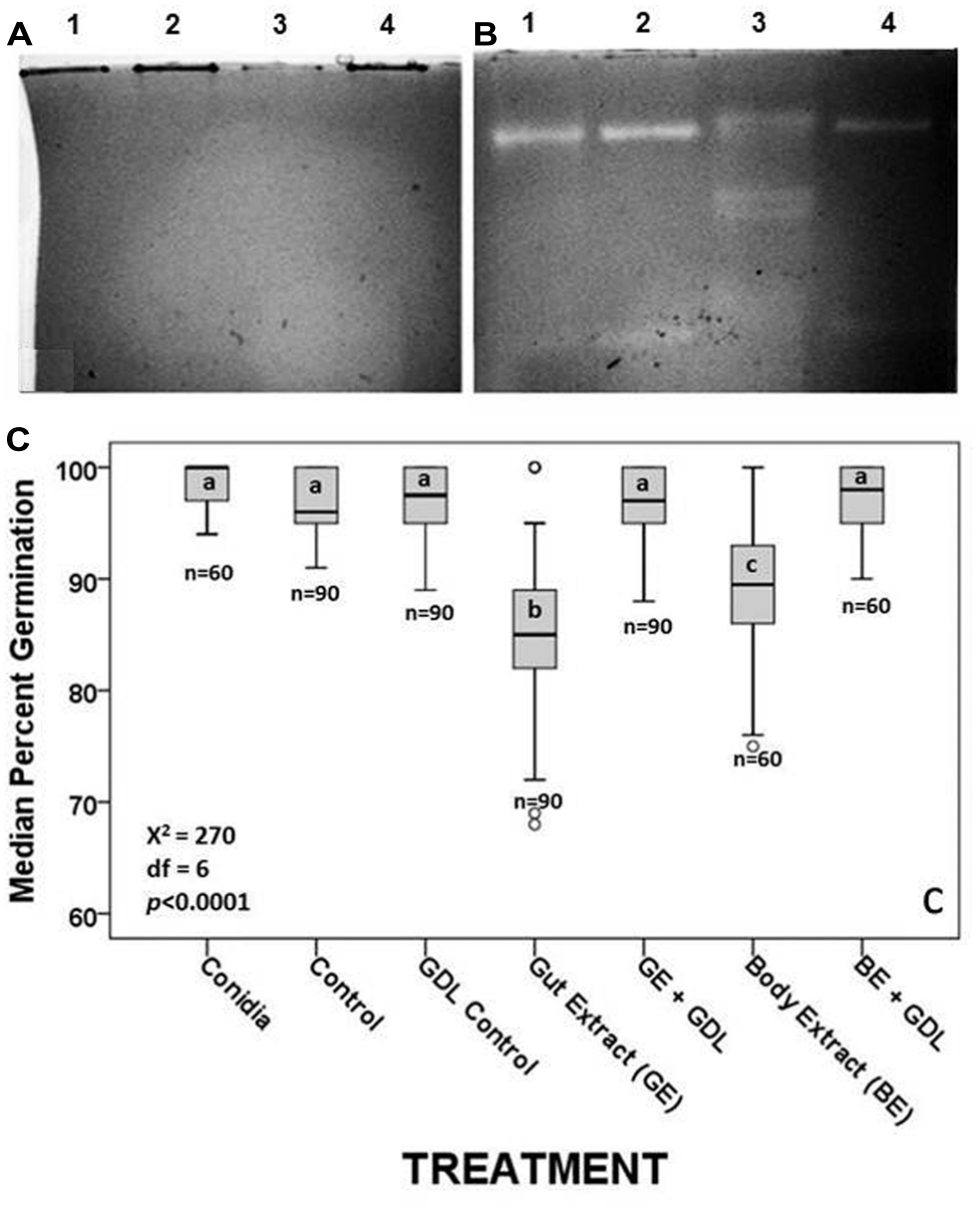
**(A)** Inactivation of β-1,3GLUs in the presence of 100 mM of the inhibitor GDL relative to the same samples without GDL **(B)**. **(C)** Median percent germination of fungal conidia following incubation with termite tissues and termite tissues treated with the inhibitor GDL. Each boxplot shows the median value and interquartile range. The outliers, identified by small circles, included cases with values between 1.5 and 3 box lengths from the upper or lower edges of the box. Bars with the same letter are not significantly different (*p* > 0.002) in pairwise comparisons (by MW test adjusted with Bonferroni correction). *n* indicates the number of field of vision on which the median germination rate was estimated.

Control samples (lacking termite extracts) were similarly prepared with 10 μL NaAc buffer, 10 μL sterile distilled water, 10 μL of ampicillin, and 10 μL of *M. anisopliae* conidia suspension (10^8^ conidia/mL) and 5 μL of GDL (100 mM). A second set of controls were set up in an identical fashion except for the GDL which was substituted by distilled water. Samples were incubated at 25°C while shaking for 6 h. Following incubation, 10 μL of the conidia-extract suspension was seeded onto a microscope slide containing a 1 mL of solidified PDA layer (*n* = 3 replicates/treatment; Rosengaus et al., 1998b). The slides were incubated for 18 h at 25°C (Supplementary Figure 3). Percent germination was estimated by counting the number of conidia with visible germ tubes (Supplementary Figure 3D) out of the total number of conidia for each of 10 fields of vision per slide [a total of 30 fields of vision (Rosengaus et al., 1998b)]. The entire experiment was replicated two or three times. We ensured that the addition of GDL inactivated the two host-derived β-1,3GLUs of the termite’s degutted body as well as the multiple β-1,3GLUs of the gut by running chromogenic gels with these samples (**Figure 3**).

Whether the *in vitro* antifungal assays were recorded as CFUs (long-lasting effects) or germination rates (early effects), fungistasis was analyzed through the use of non-parametric tests (Mann–Whitney [MW] and Kruskal–Wallis tests, SPSS, 19.0) as both measures of fungal growth (either CFUs or conidia germination) were not normally distributed. Bonferroni corrections were applied when running any multiple pairwise comparisons.

### *IN VIVO* SUSCEPTIBILITY ASSAYS

Three different colonies were used for testing whether termites with intact hindgut symbiont communities had reduced susceptibility to mycosis. Defaunated insects (by oxygenation) or pressure control (faunated) termites were allowed to walk freely for one hour inside a Petri dish (60 × 15 mm) lined with filter paper (Whatman #5) moistened with 374 μL of either a 1 × 10^5^ conidia/mL suspension of *M*. *anisopliae* or a 0.1% Tween 80 solution lacking conidia (Rosengaus et al., 1998b). In addition, a group of naïve termites (taken directly from the corresponding colonies) were also exposed to a Tween 80 or a 1 × 10^5^ conidia/mL suspension. These naïve groups were established to determine the effects that the additional manipulations resulting from the oxygenation and/or pressurization, may have had on the insect’s survival beyond those due to fungal infection alone. Termites were maintained in groups of ten during exposure to conidia or Tween 80 suspensions. Following exposure, they were transferred in these same groups to sterile Petri dishes (60 mm × 15 mm) lined with clean filter paper (Whatman # 1) and given water *ad libitum* and maintained at 25°C. The measured parameters included, survival distributions, percent survival at the end of the census, the median survival time [LT_50_; Kaplan-Meir (KM) Test] and the relative hazard ratio of death (Cox Proportional Hazard Regression Analysis). Dead termites were removed daily; surface sterilized with hypochlorite (6.0%), and plated on (PDA) plates to confirm cause of death (Rosengaus et al., 1998b). Survival analyses [Kaplan Meir (KM) tests] and a Cox proportional regression model (SPSS, 19.0) were used to estimate the time course of survival, median survival times, and relative hazard ratios of death as a function of treatment.

### ONTOGENY OF β-1,3GLUs EXPRESSION

To establish the time frame during termite development at which the expression of β-1,3GLUs begins, we created extracts of termite embryos (fertilized eggs) as well as first and second instar larvae by following the same protocols described earlier. Given their small size, these young larvae were not degutted. Instead, the entire animal (with its gut) was processed to create the extracts. The extracts were subsequently loaded onto chromogenic gels.

### SURVEY OF THE PRESENCE OF β-1,3GLUs ACROSS TERMITE SPECIES

To compare profiles of β-1,3GLUs in the body and gut tissues across species spanning the termite phylogeny, we loaded side-by-side extract samples of termites representing different species with differing nesting, feeding, and foraging habits (Abe, 1987) of the families Termopsidae (*Z. angusticollis*), Rhinotermitidae (*Reticulitermes flavipes*), Kalotermitidae (*Cryptotermes secundus*), and Termitidae (*Nasutitermes corniger*).

## RESULTS

*Zootermopsis angusticollis* have two host-derived active β-1,3GLUs in their bodies (**Figure 1A**). Additionally, they possess several extra β-1,3GLUs in their gut contents and liquid feces (**Figure 1A**). Such multiple β-1,3GLUs were observed in the hindgut region of the termite’s alimentary canal (**Figure 1B**), the same site where most symbiotic protozoa and their associated ecto- and endo-symbionts reside (Brune, 2014). In sharp contrast, the fore- and mid-gut regions of the same termites lacked the multiple clearing zones and instead only exhibited the same two β-1,3GLUs as those observed in the degutted body ((**Figures 1A,B**). Furthermore, hindgut fractions separated by centrifugation (Supplementary Figure 1) and analyzed in chromogenic gels indicated that the highest β-1,3GLUs signature (brightest clearing zones) were in the protozoa (and its associated bacteria) samples (**Figure 1C**). In comparison, the fractions containing free-living bacteria as well as the cell free supernatant had negligible β-1,3GLU activity (**Figure 1C**). That the washed gut tissue showed some enzymatic activity suggests that the protozoa and associated bacteria consortia were not completely flushed out of the tissue during the wash. Otherwise, we would have only observed the two termite-derived β-1,3GLUs (as seen in **Figure 1C**). Twelve-day-old *ex vivo* protozoa cultures of *Z. angusticollis* hindgut, consisting of mostly *Trichomitopsis termopsidis* (and their associated bacteria), also had β-1,3GLU activity which approximated that of the multiple clearing zones of faunated termite hindguts (**Figure 1D**). The differences in these β-1,3GLUs profiles likely reflect differences in the diversity and/or density of protozoa (and associated bacteria) between the *ex vivo* cultures and the *in vivo* samples. Finally, in the absence of the normal hindgut microbial community (following oxygenation treatments which destroys the anaerobic gut symbionts; Supplementary Figure 2), there was a consistent loss of the multiple β-1,3GLUs activity while the two β-1,3GLUs of bodies from the same oxygenated insects remained active (**Figure 1E**). Based on these multiple lines of evidence we are confident that the observed multiple β-1,3GLUs in the termite hindguts are of protozoa origin and/or their associated bacteria.

### *IN VITRO* ANTIFUNGAL ASSAY

Our results indicate that incubation of fungal conidia with either entire gut or degutted body extracts from faunated termites reduced fungal growth after 96 h form plating (**Figure 2A**). The degutted body extract reduced the median number of CFUs in all colonies except in colony D; the reduction in CFUs between degutted bodies and the corresponding controls was significant in colonies B and C (**Figure 2A**; MW = 6.0, *p* = 0.011; MW = 21.0, *p* = 0.003, respectively). In all six termite colonies examined, gut extracts significantly reduced fungal viability relative to the conidia alone. Furthermore, in four out of six colonies, the fungistatic effects of the faunated gut extracts were significantly greater than the inhibition caused by the degutted body (**Figure 2A**).

To test if the gut symbionts played a role in the fungistatic nature of the gut, the extracts of defaunated termites were also incubated with fungal conidia. If symbionts produce the fungistatic metabolite, removal of the symbionts (and therefore removal of their functional β-1, 3GLUs, as demonstrated in lane 1 of **Figure 1E** in the main text) should rescue conidia viability. To our surprise, the gut extracts of defaunated termites continued to reduce conidia viability relative to controls (**Figure 2B**). A comparison of the fungistatic nature of defaunated vs. faunated guts showed that the presence or absence of aerobic and facultative aerobic gut symbionts did not affect conidia viability (**Figure 2C**). Extracts of termites from Colony C were the only ones where faunated guts were significantly more fungistatic than the defaunated guts (**Figure 2C**, MW = 36.0, *p* = 0.037), as originally predicted. These results suggest that the two host-derived gut β-1,3GLUs (not affected by the oxygenation treatment, **Figure 1E**, main text) are sufficient to reduce conidia viability.

### *IN VITRO* ANTIFUNGAL ASSAY, RESCUE EFFECT OF D-GLUCONO1,5-LACTONE (GDL)

Given the visual confirmation of the loss of enzymatic function when gels were incubated with GDL (compare **Figures 3A,B**), we expected termite extracts/conidia suspensions to have increased conidia viability in the presence of GDL relative to conidia incubated with extracts containing functional β-1,3GLUs. The sodium acetate (NaAc) buffer itself, used in the preparation of conidia suspensions with tissue extracts had no negative effects on conidia viability relative to the conidia in 0.1% Tween 80 suspension. Neither did the addition of GDL or the combination of NaAC and GDL (**Figure 3C**). Therefore, we conclude that the multiple functional β-1,3GLUs of hindgut extracts were responsible for reducing germination rates by approximately 20%. Conidia incubated with the degutted body extracts containing the two host-derived β-1,3GLUs reduced conidia germination by ∼12% relative to conidia alone, conidia with NaAc, conidia with GDL and conidia with both NaAC and GDL, all of which had median percent germination of 100 (**Figure 3C**). That conidia germination rates of the different controls (conida + NaAC, conidia + GDL, conidia + NaAC + GDL) were equivalent to those of conidia incubated with GDL-inhibited termite tissue extracts demonstrates that the antifungal activity in the hindgut was indeed due to the presence of both host- and symbiont-derived functional β-1,3GLUs and that these enzymes affect fungal growth at the initial stages of fungus development (i.e., conidia germination).

### *IN VIVO* SUSCEPTIBILITY ASSAYS

**FIGURE 4:**
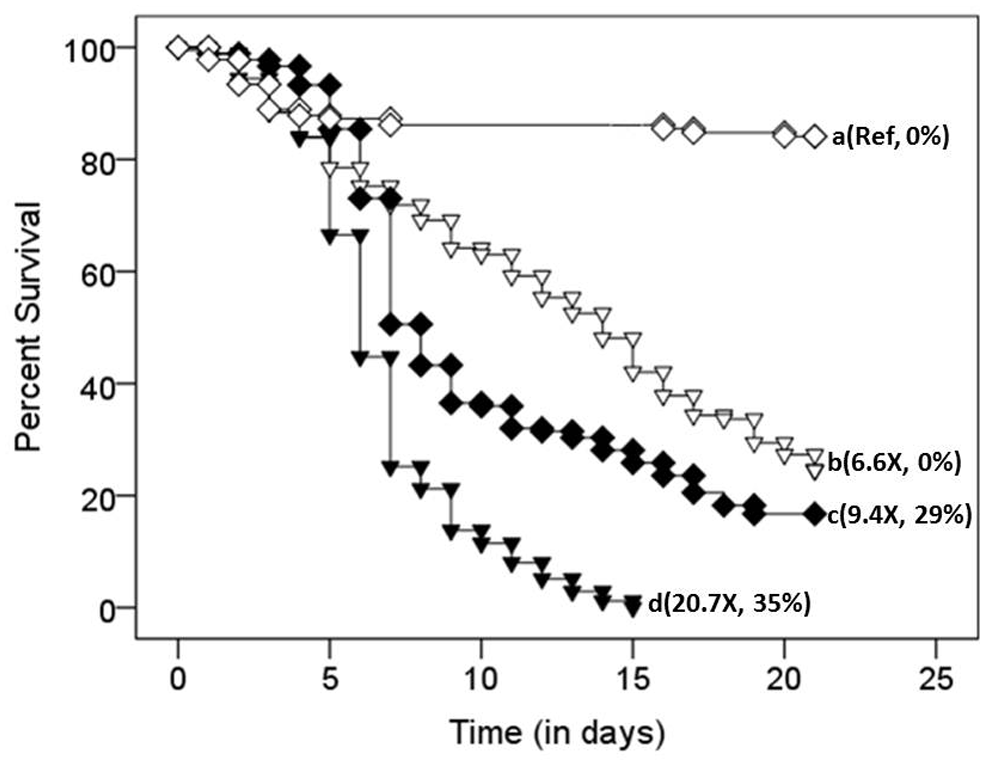
**Survival distributions of defaunated (inverted triangles) and faunated (diamonds) termites exposed to either a 0.1**%** Tween 80 solution lacking fungal conidia (open symbols) or a **0.1%** Tween 80 suspension containing 1 × 10^**5**^ conidia/mL (filled symbols).** Different letters (at the right of each survival curve) indicate significant differences (after a Bonferroni correction; *p* < 0.0001) in the time course of survival (i.e., survival distributions; by Log Rank Test, Survival Analysis). Numbers in parenthesis represent (relative hazard ratio of death, percent confirmation of *Metarhizium anisopliae* infection). Ref indicates the reference treatment against which all other hazard ratios of death were compared to.

A Cox proportional regression model showed that the colony of origin from which termites originated was not a significant and independent predictor of termite survival [Wald statistic (WS) = 4.9, df = 2, *p* > 0.05]. Hence, data from all three colonies were pooled for further statistical analyses. Treatment [faunated (pressurized only) or defaunated (pressurized and oxygenated)], on the other hand, was a significant and independent predictor of termite survival (WS = 227.4, df = 3, *p* < 0.0001). Pairwise comparisons of the combined effect of treatment and conidia exposure (Tween 80 only or Tween 80 + conidia) revealed significant differences in the time course of survival (i.e., survival distributions) between all groups [faunated controls (open diamond), faunated termites exposed to 10^5^ conidia suspension (filled diamond), defaunated controls (open inverted triangle) and defaunated termites exposed to 10^5^ conidia suspension (filled inverted triangle); **Figure 4**; KM test]. Defaunated controls experienced significantly higher mortality than faunated controls [Log Rank (LR) X^2^ = 107.1, *p* < 0.0001, **Figure 4**; KM test]. The combination of defaunation and fungal exposure significantly increased termite mortality relative to fungal exposure with pressure treatment alone (LR X^2^ = 62.2, *p* < 0.0001; **Figure 4**; KM test). Hence, in the face of pathogenic pressures, oxygenation (and its resulting defaunation) had an added effect beyond the stressors of handling and pressurization, resulting in a 27.5% difference in survival by the end of the census period. The defaunated control termites had 6.6 times the hazard ratio of death, but no confirmations of mycosis relative to faunated controls (WS = 1.9, df = 1, *p* < 0.0001). The defaunated fungal-exposed termites had the highest hazard ratio of death (20.7 times) relative to the faunated control treatment (WS = 3.0, df = 1, *p* < 0.0001) with 35% confirmation rates. Fungal-exposed faunated termites had a hazard ratio of death 9.4 times that of their control counterparts (WS = 2.2, df = 1, *p* < 0.0001), with 29% of the deaths confirmed for *M. anisopliae.*

### ONTOGENY OF β-1,3GLUs EXPRESSION

Individual embryos (fertilized eggs) had undetectable levels of β-1,3GLUs when assayed in chromogenic gels. To reach a minimal (yet noticeable) β-1,3GLU signature, at least 10 pooled embryos were required, resulting in one faint clearing zone (Supplementary Figure 4A). Extracts containing five or 10 pooled first or second instar larvae also showed detectable β-1,3GLU activity (Supplementary Figures 4A,B). Interestingly, β-1,3GLU activity of extracts containing first instar larvae were highly variable, with some samples showing little evidence while others clearly demonstrating multiple clearing zones (Supplementary Figures 4A,B). The variation across extract samples of second instar larvae was reduced relative to that of first instar larvae.

### SURVEY OF THE PRESENCE OF β-1,3GLUs ACROSS TERMITE SPECIES

To compare profiles of β-1,3GLUs in the body and gut tissues across species spanning the termite phylogeny, we loaded side-by-side extract samples of termites representing different families [Termopsidae: *Z. angusticollis (Z.a.)],* [Rhinotermitidae: *R. flavipes (R.f.*)], [Kalotermitidae: *C. secundus (C.s.*)] and families [Termitidae: *N. corniger (N.c.)*; Supplementary Figure 5]. While all species exhibited two termite-derived β-1,3GLUs in their body (minus the gut) tissue, these enzymes were not identical given their different migration patterns in the gel. Interestingly, the fact that dissected guts of the “higher” termite *N. corniger* (Bulmer et al., 2009; Supplementary Figure 5) have only two β-1,3GLUs provides further support to our conclusion that the source of these multiple hindgut enzymes of termites are the protozoa and their associated bacteria. *N.c.* lack the typical hindgut protozoa communities of the “lower” termites (Ohkuma and Brune, 2011).

## DISCUSSION

Through a combination of chromogenic gels, tissue dissections and fractionation, *ex vivo* culturing of termite hindgut symbionts and defaunation experiments, we provide, for the first time, evidence that the multiple functionally active β-1,3GLUs in *Z. angusticollis* hindguts are synthesized by protozoa and/or their associated bacteria. In contrast, the two β-1,3GLUs found in tissues other than the hindgut are likely of termite-origin. This is supported by data from the recent *Z. nevadensis* genome (Terrapon et al., 2014) which shows that out of the six Gram-negative binding proteins (GNBPs), two correspond to β-1,3GLUs identified in other termites (Hamilton et al., 2011; Gao et al., 2012; Hamilton and Bulmer, 2012; Hussain et al., 2013) and *Cryptocercus* wood cockroaches (Bulmer et al., 2012).

It remains to be determined whether the additional β-1,3GLUs’ of the termite’s hindgut are synthesized by the protozoa themselves or by their associated bacteria. Many bacteria are intimately associated with the protozoa either on their surface or as intracellular endo-symbionts (Brune, 2006, 2014; Strassert et al., 2012) and potentially, they too could be the source of the multiple β-1,3GLUs. Unfortunately, because of their inherent co-dependency, the experimental elimination of bacterial ecto- and endo-symbionts also negatively affected the protozoa population. Hence, further studies are needed to identify which microbes in the protozoa/bacteria partnership are responsible for the synthesis of β-1,3GLUs.

Our results also point to the likelihood that both the termite- and symbiont-derived β-1,3GLUs have fungistatic activity (**Figure 2**). Such an effect is likely due to the enzymes’ ability to break β-1,3 glycosidic linkages of β-1,3 glucans, the principal component of fungal cell walls (Brown and Gordon, 2005). We have previously demonstrated that β-1,3GLUs cause *Metarhizium* conidia to collapse, decreasing conidia volume by 25% (Bulmer et al., 2009; Rosengaus et al., 2011). The possibility exist that such conidia shrinkage resulted from using natural and commercially available β-1,3GLUs at concentrations above the physiological concentrations normally used by termites. Nevertheless, these results indicate that β-1,3GLUs can damage and affect both conidia integrity and its germination, resulting in prolonged fungistasis (at least at 96 h post-plating).

The possibility exists that compounds other than (or in combination with) the multiple β-1,3GLUs, including termicin (Hamilton and Bulmer, 2012), may be responsible for the refractive nature of termite guts against fungus growth (Siderhurst et al., 2005; Chouvenc et al., 2008, 2009, 2010). The loss of activity of β-1,3GLUs through the addition of GDL, a competitive inhibitor, and its accompanying rescue effects on conidia viability (**Figure 3**) together with our observation that body alone (minus the gut) and gut extracts have similar fungistatic properties (**Figure 2**) provide indirect evidence that β-1,3GLUs can help control fungal germination. Our *in vivo* data also suggest that termites with an intact gut microbial community are less susceptible to mycosis. Regrettably, because immune defenses and susceptibility to mycosis are probably influenced by nutritional status, we cannot pull apart the independent effects of these coupled factors. Yet, in the face of pathogenic pressures, oxygenation (and its resulting defaunation) had an added effect beyond the stressors of handling and pressurization, particularly within the first 10 days of the census period (**Figure 4**), when the effects on malnourishment (due to the lack of gut symbionts) should have had lower impacts on survival. Ultimately, by day 20 post-exposure to fungal conidia, there was a 27.5% difference in the survival of defaunated (oxygenated) and faunated (pressurized) termites. Results from other termite species demonstrate that GDL-treated insects (i.e., with inhibited β-1,3GLUs’) are more susceptible to mycosis (Bulmer et al., 2009; Hamilton et al., 2011; Hamilton and Bulmer, 2012). Taken together, all these results strongly suggest that the functional symbiont-derived β-1,3GLUs play some role in the control of mycosis.

It is unlikely that the gut’s hostile environment toward conidia germination in previous (Chouvenc et al., 2009, 2010) and our current experiments was due to factors such as gut pH or low oxygen levels. The pH of the termite’s paunch, the hindgut region where the protozoa and their ecto- and endosymbionts reside, is within the range of *M. anisopliae* germination (Dillon and Charnley, 1986; Brune et al., 1995). Furthermore, while an oxygen deficit is normally inhibitory to the germination of conidia (Cochrane et al., 1963), the termite gut is not completely anoxic as the perimeter regions of the alimentary canal (where conidia would likely be germinating) are oxic (Ebert and Brune, 1997). Remarkably, the fungistatic effects of termite guts persist even after termites die (Chouvenc et al., 2009) suggesting that whichever antifungal compound(s) exist in the termite’s guts, they are environmentally stable and not easily degradable. These same traits are characteristic of the multiple hindgut β-1,3GLUs which retain robust activity long after their incorporation into the termite’s nest structure (Bulmer et al., 2009; Hamilton et al., 2011; Hamilton and Bulmer, 2012). Although the gut microbiota of lower termites has been historically associated with cellulose degradation and nitrogen fixation (Scharf et al., 2011; Brune, 2014), our results suggest that their function may extend beyond nutrition. In conjunction with other factors, the gut microbiome may also provide protection against mycosis.

Notably, the putative protective role of the multiple hindgut β-1,3GLUs may extend beyond the individual termites. The benefits derived from harboring an intact gut microbiome (along with the functional β-1,3GLUs) can be also appreciated at the communal level. Because termites use their liquid and solid feces in nest construction (Rosengaus et al., 1998b) and given that the liquid feces have β-1,3GLU activity [**Figure 1A**, but not the fecal pellets (Rosengaus et al., 2013)], the excretion and incorporation of β-1,3GLUs from the liquid feces into the termite’s nest structure likely expands mycosis protection to the entire colony. Although previous research has demonstrated that termite feces have potent antifungal properties (Rosengaus et al., 1998b; Chouvenc et al., 2013) and that Actinobacteria may be responsible for such negative effects (Chouvenc et al., 2013), no information exists on whether microbes colonizing the termite fecal pellets produce β-1,3GLUs. Similarly, at the communal level, the active β-1,3GLU secreted by the salivary glands of some termites (Bulmer et al., 2009; Hamilton et al., 2011), in conjunction with termicin (Lamberty et al., 2001), may be playing an antifungal role when these compounds are smeared onto the cuticle of nestmates during mutual grooming. The combined effects of performing social interactions and the deposition of biochemical secretions with antifungal properties (i.e., termicins and β-1,3GLUs) likely increase resistance against mycosis throughout the entire colony.

Embryos have one β-1,3GLU, albeit in extremely low quantities. This suggests that the expression of at least one host-derived β-1,3GLU starts early during the termite’s development. Given that liquid feces of *Z. angusticollis* have identical β-1,3GLUs profiles as those from faunated hindguts (**Figure 1A**), and that first and second instar larvae exhibit the typical multiple symbiont-derived β-1,3GLUs, we are confident that these young individuals acquire the additional enzymes along with the full complement of hindgut microbes through common proctodeal exchanges (anus-to-mouth feeding; Supplementary Figure 4). These exchanges ensure that young nestmates benefit not only from attaining cellulolytic activity and nitrogen supplementation (Machida et al., 2001), but also from protection against pathogenic fungi during the insects’ development. If the hindgut β-1,3GLUs of other lower termites (Bulmer et al., 2010; Hamilton et al., 2011; Hamilton and Bulmer, 2012) are also of microbial origin and are similarly shared among nestmates via proctodeal exchanges, this and the work by Koch and Schmid-Hempel (2011) may represent prime examples of widespread symbiont-mediated social immunity across the termites (Traniello et al., 2002; Cremer et al., 2007; Rosengaus et al., 2011). Termites, and their microbiome, are therefore excellent test organisms to address ecological immunology questions against the background of eusociality.

The mutualistic association with hindgut microbes that synthesize β-1,3GLUs was likely established before the evolution of termites. *Cryptocercus punctulatus,* the closest extant roach relative of termites (Nalepa and Bandi, 2000; Lo and Eggleton, 2011), also contains β-1,3GLU activity in its hindgut (Bulmer et al., 2012) and its liquid feces (Rosengaus et al., 2013). They also share many of the same gut microbiota with termites (Brune, 2014). The most parsimonious explanation for the presence of multiple β-1,3GLUs in both *Cryptocercus* and “lower” termites’ hindguts as well as their liquid feces, is that their common ancestor already possessed a microbial community capable of synthesizing β-1,3GLUs, which could be then shared among family members via proctodeal exchanges. The presence of such enzymes could have served as an important pre-adaptation for the evolution of group-living as family units of *Cryptocercus* and early termite-like ancestors likely exhibited enhanced resistance against pathogenic fungi while nesting in and feeding on decayed wood even before the inception of eusociality (Rosengaus et al., 2011). Based on the few termite species tested, the existence of multiple hindgut β-1,3GLUs appears to be conserved across the “lower” termites (Bulmer et al., 2010; Hamilton et al., 2011), although given their different migration patterns in gels (Supplementary Figure 5), these enzymes likely underwent an independent diversification within each termite lineage.

## CONCLUSION

Our data show that the multiple β-1,3GLUs in *Z. angusticollis* are synthesized by the hindgut protozoa (and/or its associated bacteria). The presence of such enzymes may help explain in part, why the termite’s alimentary canal appears hostile to fungal development. Additional *in vivo* experiments indicate that termites with an intact hindgut microbiota (and its accompanying β-1,3GLUs) are less susceptibility to mycosis. Through proctodeal feedings, termites effectively share and distribute their hindgut microbial consortia along with their fungistatic activity, a prime example of symbiont-mediated social immunity. Studying the holobiont in general, and the microbiome’s impacts on host resistance to disease in particular, is critical in understanding the role that microorganisms play in the evolution of important ecologically relevant host traits (Margulis and Fester, 1991; Feldhaar, 2011), including perhaps the evolution of insect eusociality.

## AUTHOR CONTRIBUTIONS

Rebeca B. Rosengaus, Kelley F. Schultheis, Mark S. Bulmer participated in all aspects of this research. Alla Yalonetskaya ran the defaunation and *in vivo* susceptibility assays while William S. DuComb, Ryan W. Benson, and Veronica Godoy-Carter carried out the chromogenic gel separation for GDL experiments. John P. Thottam and Rebeca B. Rosengaus performed the antifungal activity assays using the GDL inhibitor. Rebeca B. Rosengaus funded the work while Rebeca B. Rosengaus, Kelley F. Schultheis, Mark S. Bulmer, and Veronica Godoy-Carter wrote the manuscript.

## Conflict of Interest Statement

The authors declare that the research was conducted in the absence of any commercial or financial relationships that could be construed as a potential conflict of interest.
