# Supplementary material for: Symbiont-derived β-1,3-glucanases in a social insect: mutualism beyond nutrition
Source: Front Microbiol. 2014 Nov 21;5:607. doi: 10.3389/fmicb.2014.00607 (PMC4240165; doi:10.3389/fmicb.2014.00607)
Supplement: Supplementary file 1 [file Data_Sheet_1.PDF]

Electronic Supplementary Material
Frontiers in Microbiology
Microbial Symbioses
Original Research Article

Symbiont-derived *β*-1,3-glucanases in a social insect: mutualism beyond nutrition

Rebeca B. Rosengaus[1*], Kelley F. Schultheis[1], Alla Yalonetskaya[1], Mark S. Bulmer[2], William S. DuComb[3], Ryan W. Benson[3,‡], John Paul Thottam[1], Veronica Godoy-Carter[3].

[1] Department of Marine and Environmental Sciences, Northeastern University, Boston, MA, USA

[2] Department of Biological Sciences, Towson University, Towson, MD, USA.

[3] Department of Biology, Northeastern University, Boston, MA, USA

Correspondence
Department of Marine and Environmental Sciences
Northeastern University
134 Mugar Life Sciences Building
360 Huntington Avenue
Boston, MA 02115-5000
r.rosengaus@neu.edu

[‡]Current address: Forsyth Institute, Cambridge, MA, USA

Keywords: gut protozoa, disease resistance, *β*-1,3-glucanases, termites, social immunity, mycosis

Preparation of gut fractions via centrifugation

A detailed description of the centrifugation protocol is included in the main text of the article. **Supplementary Figure 1** should be seen in conjunction with such detailed description and **Figure 1C** in the main article.

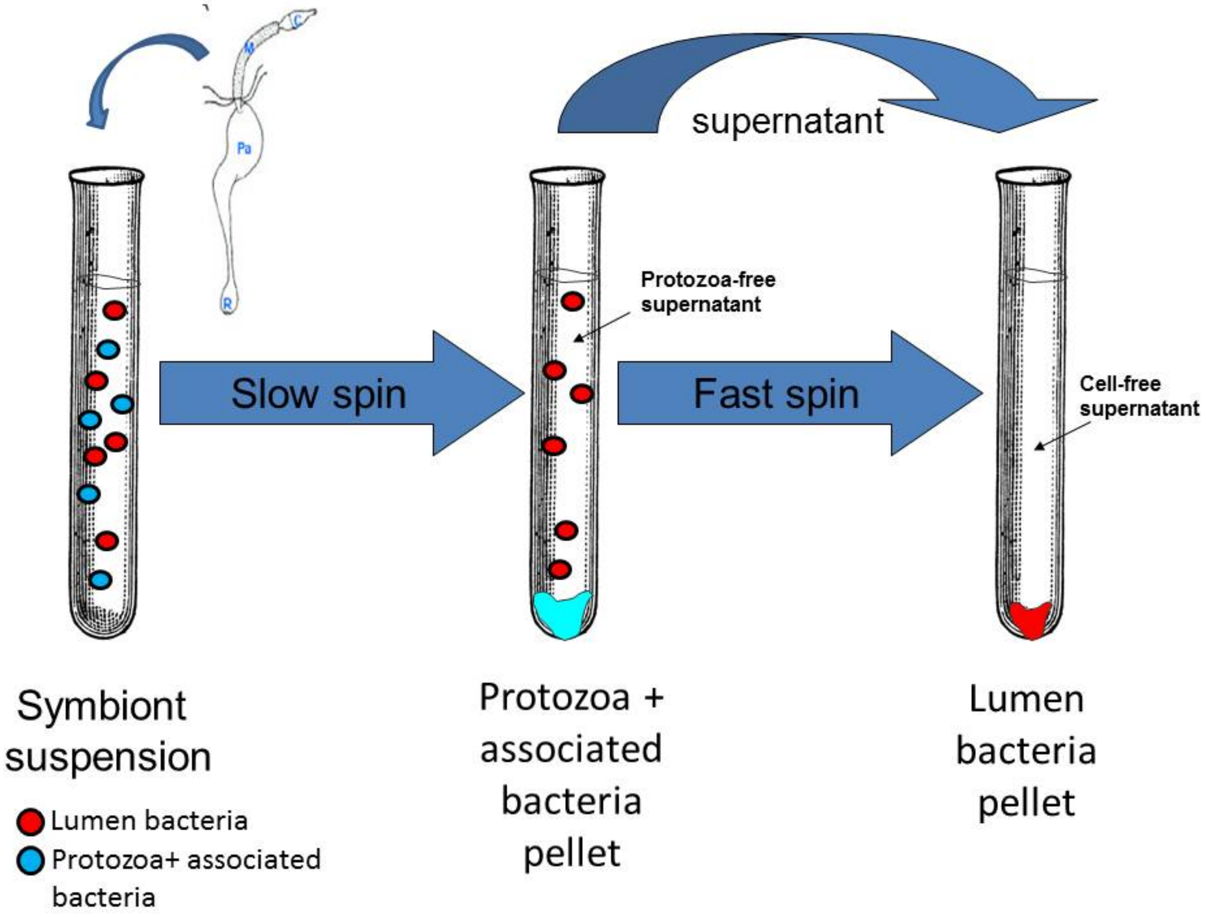

Supplementary Figure 1. Centrifugation protocol to separate different fractions which were then loaded onto CM Curdlan RBB gel (**Figure 1C**, main article). C = crop of the termite's digestive system, M = midgut, P = paunch (hindgut), R = rectum.

Defaunation Procedure

To confirm that the oxygenation procedure (n = 12) resulted in the defaunation of termites relative to termites in the control (pressurized animals without added oxygen, n = 12) and naïve animals (untreated n = 7), 10 μL of hindgut fluid were examined for the presence/absence and number of intact protozoa post-treatment. The number of intact protozoa/mL/termite was quantified using a compound light microscope (Nikon Eclipse E400) at 400 x magnification. All defaunated termites had zero intact protozoa regardless of colony of origin. Our results confirmed that the oxygenation procedure was effective in ridding termites from their hindgut protozoa community, and that pressurization (without oxygenation) had no effect on the termite's gut microbiome (**Supplementary Figure 2**).

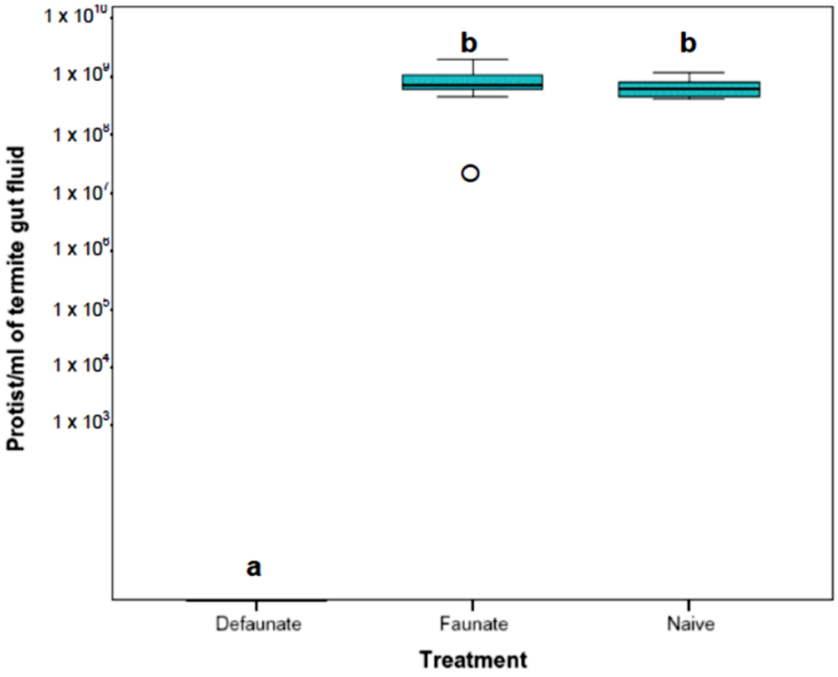

Supplementary Figure 2. Number of protists/mL of termite gut fluid for defaunated termites faunated control termites and naïve termites The remaining boxplots show the median value and interquartile range. Outliers, identified by an open circle, included cases with values between 1.5 and 3 box lengths from the upper edge of the box. Bars with the same letter are not significantly different ($p > 0.017$) in pairwise comparisons (by MW test adjusted with Bonferroni correction, SPSS 19.0).

Long- and short-term effects of *β*-1,3GLUs on conidia viability

We used two different methods to establish the effects of *β*-1,3GLUs on conidia viability. The long-term effects were calculated using the Colony Forming Units (CFUs) technique where CFUs were quantified after 96 hours from plating. Control conidia plated on potato dextrtose agar (**Supplementary Figure 3A**) had significantly more CFUs than conidia incubated with extracts containing active *β*-1,3GLUs (**Supplementary Figure 3B**). The short-term effects of *β*-1,3GLUs were quantified by estimating the percent germination rates 18 hours post-plating. **Supplementary Figure 3C** depicts un-germinated conidia from the control treatment while **Supplementary Figure 3D** shows the high germination rates of these control conidia. Conidia incubated with termite extracts and their accompanying *β*-1,3GLUs (not shown) had a significantly higher proportion of un-germinated conidia than controls (**Supplementary Figure 3D**).

CFUs enumerated at 96 hours post-plating | **Germination rates quantified at 18 hours post-plating**

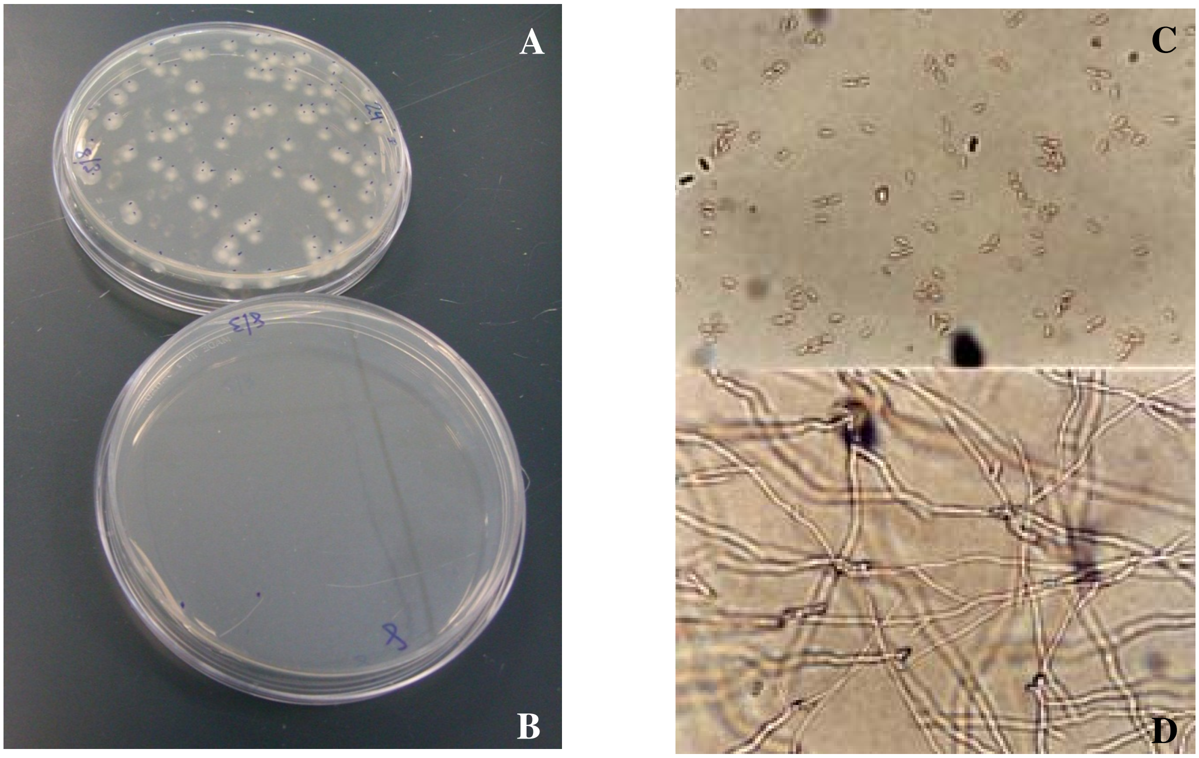

Supplementary Figure 3. Results of *in vitro* experiments testing the long-lived and early effects of *β*-1,3GLUs on fungal growth using the CFU (**Supplementary Figure 3A, B**) and conidia germination protocols (**Supplementary Figure 3C, D).**

Ontogeny of *β*-1,3GLUs Activity

The detection of functional *β*-1,3GLUs was determined for fertilized eggs (embryos), first and second instar larvae. Protocol details in the text of main article.

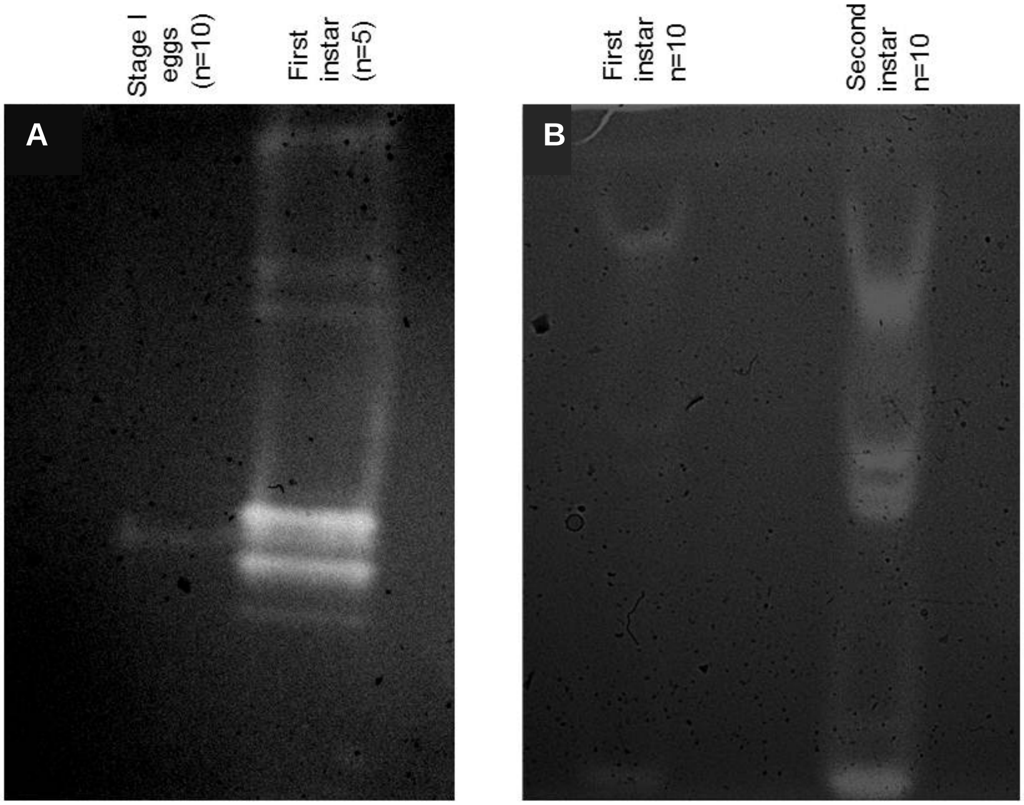

Supplementary Figure 4. *β*-1,3GLUs in liquid feces (**Figure 1A** in main article) approximates the *β*-1,3GLUs profile of first and second instar larvae following transfaunation by proctodeal (anus-to-mouth) feedings (A, B). Note that 10 pooled eggs have scarcely detectable *β*-1,3GLU activity (A). First instar larvae lack the microbial gut consortia at hatching. They likely acquire the hindgut microbiota (along with the functional *β*-1,3GLU) after transfaunation by their older nestmates. Hence, through proctodeal exchanges, young larvae acquire not only the microbial communities for cellulose digestion, but also the *β*-1,3GLUs that may protect them against mycosis.

Presence of β-1,3GLUs Across Termite Phylogeny

To compare profiles of β-1,3GLUs in the body and gut tissues across species spanning the termite phylogeny, we loaded side-by-side extract samples of termites representing basal *(Zootermopsis angusticollis [Z.a.]),* intermediate *(Reticulitermes flavipes [R.f.]* and *Cryptotemes secundus [C.s.])* and derived families *(Nasutitermes corniger [N.c.]*; **Supplementary Figure 5).** While all species exhibited two endogenous β-1,3GLUs in their body (minus the gut) tissue, these enzymes were not identical given their different migration patterns in the gel. Interestingly, the fact that dissected guts of the derived *N. corniger* (Bulmer et al., 2009; **Supplementary Figure 5)** have only two β-1,3GLUs provides further support to our conclusion that the source of these multiple hindgut enzymes of basal termites are the protozoa and their associated bacteria. *N.c.* lack the typical hindgut protozoa communities of the "lower" termites (Ohkuma and Brune, 2011).

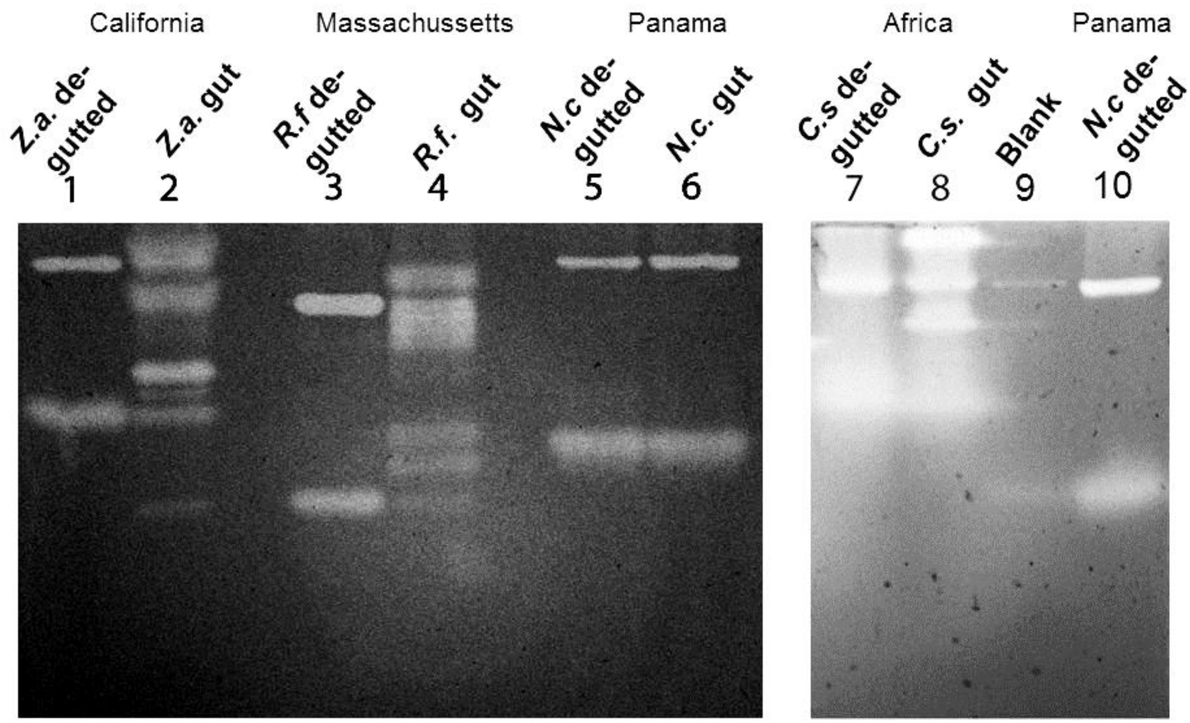

Supplementary Figure 5. Side-by-side β-1,3GLU profiles in the body and gut tissues of different termite species. *Cryptotermes secundus* samples were kindly provided by Dr. Judith Korb. Similar β-1,3GLUs activity for *Nasutitermes corniger* and *Reticulitermes flavipes* has been reported by (Bulmer et al., 2009; Hamilton and Bulmer, 2012; Hamilton et al., 2011). Note that although lane 9 was blank, extract spill-over from adjacent lanes may have occurred.

References

Bulmer, M.S., Bachelet, I., Raman, R., Rosengaus, R.B., and Sasisekharan, R. (2009). Targeting an antimicrobial effector function in insect immunity as a pest control strategy. *Proc. Natl. Acad. Sci. U.S.A.* 106**,** 12652-12657.

Hamilton, C., and Bulmer, M.S. (2012). Molecular antifungal defenses in subterranean termites: RNA interference reveals in vivo roles of termicins and GNBPs against a naturally encountered pathogen. *Dev. Comp. Immunol.* 36**,** 372–377.

Hamilton, C., Lay, F., and Bulmer, M.S. (2011). Subterranean termite prophylactic secretions and external antifungal defenses. *J. Insect Physiol.* 57**,** 1259-1266.

Ohkuma, M., and Brune, A. (2011). "Diversity, structure, and evolution of the tertmite gut microbial community," in *Biology of Termites: a Modern Synthesis* eds. D.E. Bignell, Y. Roisin & N. Lo. (Berlin: Springer-Verlag), 413-438.

Rosengaus, R.B., Guldin, M.R., and Traniello, J.F.A. (1998). Inhibitory effect of termite fecal pellets on fungal spore germination. *J. Chem. Ecol.* 24**,** 1697-1706.
